# Warming and drought weaken the carbon sink capacity of an endangered paleoendemic temperate rainforest in South America

**DOI:** 10.1029/2022JG007258

**Published:** 2023-04-03

**Authors:** Jorge F. Perez-Quezada, Jonathan Barichivich, Rocío Urrutia-Jalabert, Enrique Carrasco, David Aguilera, Cédric Bacour, Antonio Lara

**Affiliations:** 1 Department of Environmental Science and Renewable Natural Resources, University of Chile, Avenida Santa Rosa 11315, Santiago, Chile; 2 Institute of Ecology and Biodiversity, Victoria 631, Barrio Universitario, Concepción, Chile; 3 Cape Horn International Institute, Ave. Bulnes 01855, Punta Arenas, Chile; 4 Laboratorio de Dendrocronología y Cambio Global, Instituto de Conservación, Biodiversidad y Territorio, Facultad de Ciencias Forestales y Recursos Naturales, Universidad Austral de Chile, Valdivia, Chile; 5 Laboratoire des Sciences du Climat et de l’Environnement (LSCE), LSCE/IPSL, CEA-CNRS-UVSQ, Université Paris-Saclay, Gif-sur-Yvette, France; 6 Departamento de Ciencias Naturales y Tecnología, Universidad de Aysén, Coyhaique, Chile; 7 Center for Climate and Resilience Research (CR)2, Universidad de Chile, Santiago, Chile; 8 Fundación Centro de los Bosques Nativos FORECOS, Valdivia, Chile

**Keywords:** *Fitzroya*, carbon cycle, gross primary productivity, eddy covariance, fluorescence, environmental thresholds

## Abstract

Measurements of ecosystem carbon (C) fluxes in temperate forests are concentrated in the Northern Hemisphere, leaving the functionally diverse temperate forests in the Southern Hemisphere underrepresented. Here, we report three years (February 2018-January 2021) of C fluxes, studied with eddy-covariance and closed chamber techniques, in an endangered temperate evergreen rainforest of the long-lived paleoendemic South American conifer *Fitzroya cupressoides*. Using classification and regression trees we analyzed the most relevant drivers and thresholds of daily net ecosystem exchange (NEE) and soil respiration. The annual NEE showed that the forest was a moderate C sink during the period analyzed (-287±38 g C m^-2^ year ^-1^). We found that the capacity to capture C of the *Fitzroya* rainforests in the Coastal Range of southern Chile is optimal under cool and rainy conditions in the early austral spring (October-November) and decreases rapidly towards the summer dry season (January-February) and autumn. Although the studied forest type has a narrow geographical coverage, the gross primary productivity measured at the tower was highly representative of *Fitzroya* and other rainforests in the region. Our results suggest that C fluxes in paleoendemic cool *F. cupressoides* forests may be negatively affected by the warming and drying predicted by climate change models, reinforcing the importance of maintaining this and other long-term ecological research sites in the Southern Hemisphere.

## 1 Introduction

Forests cover around 25-30% of the Earth surface (WRI, 2001) and are thus central to the global carbon (C) cycle and mitigation of climate change (Pan et al., 2011). Major efforts for quantifying and monitoring forest-climate interactions have been dedicated across more than ~900 sites of flux towers and chamber systems around the world (FLUXNET, 2021). These monitoring sites are organized in regional (Novick et al., 2018; Yu and Hirano, 2021) and global (Bond-Lamberty, 2020; Papale, 2020) networks, which together allow understanding and modelling of the patterns and environmental controls of ecosystem carbon and water cycling in major forest biomes (Baldochi 2020). However, the spatial coverage of monitoring sites is still strongly biased towards Northern Hemisphere biomes and developed countries. As a result, the tropics and Southern Hemisphere biomes are heavily under-represented in flux networks (Pastorello et al. 2020, Villarreal and Vargas 2021). In addition, local and variable spatial footprints of flux towers (Chu et al., 2021) and chamber systems may limit the representativeness of flux measurements within biomes and heterogeneous regions.

Carbon fluxes in the temperate rainforests of South America and their environmental drivers cannot be assumed to be similar to their counterparts in the north because of substantial differences in their biogeographical histories and functional diversity (Alaback 1991; Lusk et al., 2004). Therefore, it is necessary to measure and determine how austral temperate forests behave and respond to climate change. A recent study of carbon fluxes in a North-western Patagonian evergreen forest from southern South America (42° S) showed lower net ecosystem exchange, but higher biological activity (higher gross primary productivity and ecosystem respiration) compared to rainforests in the Northern Hemisphere (Perez-Quezada et al., 2018). This same forest was also found to store the largest C stock in the soil among temperate rainforests in the world (Perez-Quezada et al., 2021).

*Fitzroya cupressoides* is an endangered paleoendemic conifer (Ahuja 2017) of the temperate rainforests of southern South America (Donoso et al., 2006; IUCN, 2021). Palaeoendemics are ancient taxonomic groups of organisms that are geographically confined to regions where ecologically important features of ancient environments survive but have been lost elsewhere (Jordan et al., 2006). It is one of the two longest-lived tree species in the world, together with bristlecone pine (*Pinus longaeva*), with ages precisely dated around 3600 years (Lara and Villalba, 1993) and a maximum age likely surpassing 5000 years (Popkin, 2022). *Fitzroya* forests are among the most carbon massive (>510 Mg C ha^-1^ of aboveground biomass) and have the slowest carbon dynamics reported for rainforests in the world (Urrutia-Jalabert et al., 2015a; González et al. 2022). Despite their slow carbon dynamics, *Fitzroya* forests act as an effective carbon sink, especially due to the exceptionally long mean residence time of their wood that can exceed 1500 years in the Andes foothills (Urrutia-Jalabert et al., 2015a). At present, most of the remaining *Fitzroya* forests occur along the Andes in southern Chile and in the mountaintops of the Coastal Range, with contrasting levels of standing biomass. Compared with record high biomass levels in the Andes (>500 Mg C ha^-1^), aboveground biomass is much lower in the Coastal Range (115 Mg C ha^-1^, Urrutia-Jalabert et al., 2015a) because of repeated fire disturbance (Barichivich, 2005; Lara et al., 1999), lower soil fertility and drier summers compared to the Andes (Urrutia-Jalabert et al., 2015a, 2015b).

*Fitzroya* forests in the northern part of the Coastal Range are the northernmost stands of the species, being likely more vulnerable to climate change. In 2018, an eddy-covariance flux tower was installed to monitor water and carbon cycling in a representative *Fitzroya* forest stand of the Coastal Range. The tower is deployed in a long-term research site at the Alerce Costero National Park (ACNP). Forests around the tower are a mosaic of secondary dense and semi-open stands established after a stand-replacing fire at the end of the 17th century (Urrutia-Jalabert et al., 2015c, 2018). A few low-intensity fires have affected some nearby forest patches located within 4 km from the eddy tower during the logging phase in the past century. Soils are thin and infertile and have low water retention capacity, thus often saturate in winter and dry up during summer (Urrutia-Jalabert et al., 2015b, 2015c). Such soil properties enhance the stress on tree growth from drought, high temperatures and high vapor pressure deficit compared to evergreen broadleaved forests growing on better soils (Urrutia-Jalabert et al., 2015b).

Tree-ring studies near the flux tower showed that the ongoing regional drying and warming trends with climate change have been causing a growth decline during recent decades (Barichivich, 2005; Urrutia-Jalabert et al., 2015c), which has not yet been seen in the Andes (Lara et al., 2020; Lavergne et al., 2018). Tree-ring carbon isotopes showed that despite this declining growth, leaf-level intrinsic water use efficiency of this forest has increased with rising CO_2_ concentrations in the atmosphere, likely due to a decrease in stomatal conductance (Urrutia-Jalabert et al., 2015c). At the ecosystem level, soil respiration in the area was found to increase substantially with warm and dry conditions during summer, reinforcing the idea that the carbon sink of *Fitzroya* rainforest ecosystems in the Coastal Range can be negatively affected by current and projected climate trends (Urrutia-Jalabert et al., 2017).

Here we present the first study that documents the ecosystem and soil carbon exchanges in the endangered *Fitzroya* forests using eddy-covariance and closed chamber techniques. Our aims were to: 1) assess the seasonal cycles of carbon capture and annual carbon balance, 2) identify the leading climatic drivers and thresholds that influence carbon fluxes, and 3) quantify the spatial representativeness of gross productivity measured at the tower in the context of the temperate rainforests in the region. We hypothesize that the forest is a net carbon sink, but this is weakened by hot and dry summers because of increased carbon respiration losses.

## 2 Materials and Methods

### 2.1 Study site and forest stand

**Figure 1:**
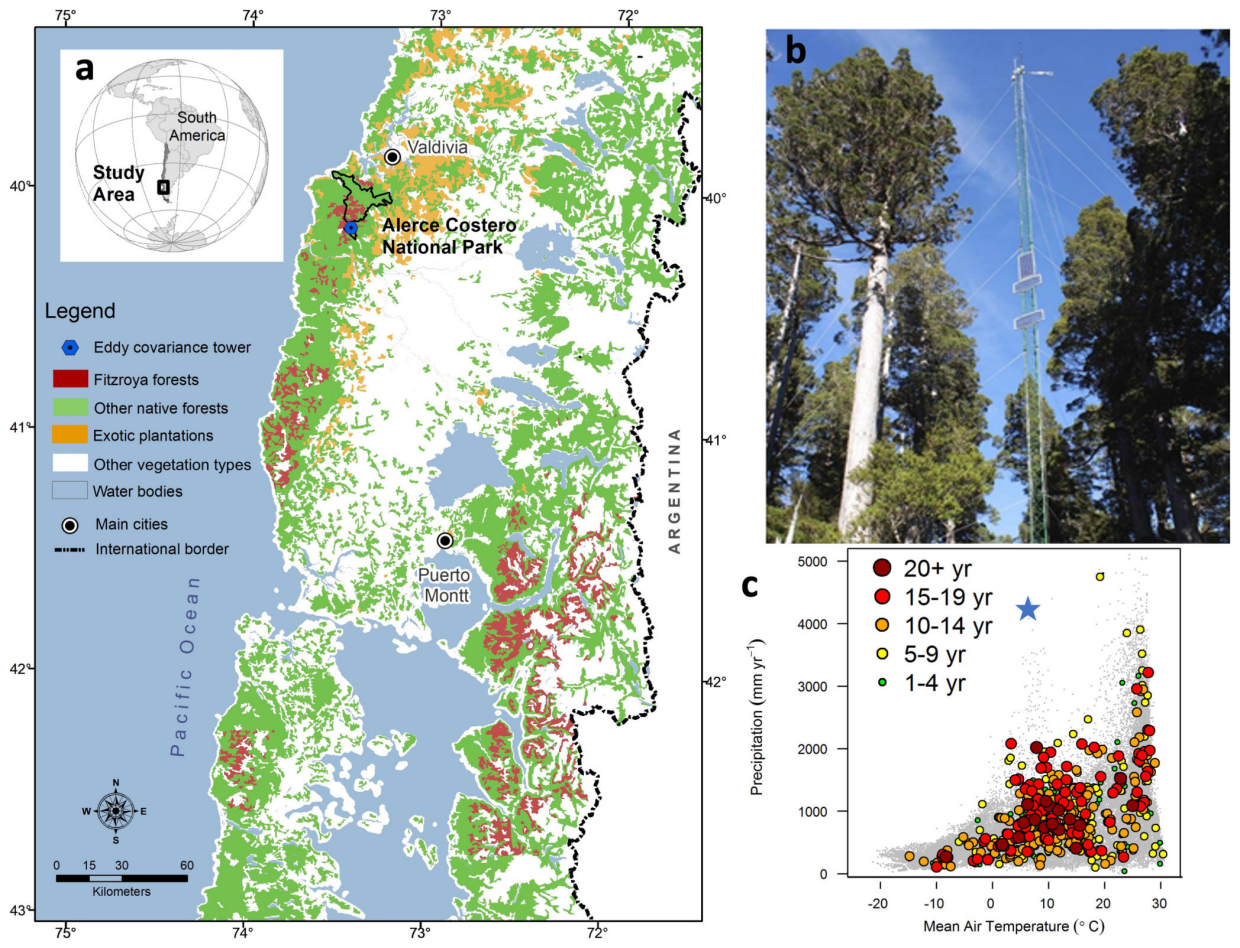
a) Distribution of *Fitzroya cupressoides* and other native forests, and location of the Alerce Costero forest eddy-covariance tower (CL-ACF); b) picture of the forest stand and the 36-m flux tower; c) position of the CL-ACF tower (blue star at 7.5°C and 4180 mm) in the temperature-precipitation climate space of the sites registered in the Fluxnet2015 Dataset. Circles are coloured depending on the number of data years available (modified from FLUXNET, 2021).

The study site corresponds to a *Fitzroya cupressoides* forest located in a flat hilltop (slope < 5%) at 850 m altitude in the Coastal Range of southern Chile, protected by the Alerce Costero National Park (40°10´ S, 73°26´ W; Figure 1). Climate in the study area is characterized by annual precipitation of 4180 mm, although the Mediterranean climate influence in the area generates very wet winters and dry summers (Urrutia-Jalabert et al., 2015b). Mean annual temperature is ~7.5 °C, with mean winter and summer temperatures of about 3.5 and 11.7 °C, respectively (Urrutia-Jalabert et al., 2015b, 2018). Soils in the area have originated from Pre-Cambrian to Paleozoic metamorphic rocks. In general, they are very shallow (depths 40-60 cm), sandy and with a very low nutrient content due to a continuous process of lixiviation, because of high precipitation. Because of these characteristics, soils have a very low water retention capacity, getting waterlogged in winter months and very dry during summer (Heusser, 1982; Urrutia-Jalabert et al., 2015b).

The forest that surrounds the flux tower is dominated by adult *Fitzroya cupressoides* trees (17-18 m tall and 40-60 cm in diameter at breast height). Accompanying tree species are mainly evergreen broadleaved: *Nothofagus nitida*, *Nothofagus betuloides*, *Drimys winteri*, *Tepualia stipularis*, *Embothrium coccineum* and *Weinmannia trichosperma* (Urrutia-Jalabert et al., 2015a). There are also very dense patches of *Fitzroya* saplings and young trees in areas where this species regenerated abundantly after fires occurred before the 1930s, with an understory dominated by evergreen species like *Chusquea nigricans*, *Gaultheria spp*., *Desfontainia fulgens*, *Ugni candollei* and the fern *Blechnum magellanicum*. The adult forest around the tower is semi-dense (canopy cover 50-75%), medium-age and likely established after a fire in 1681, with the oldest trees being around 15-18 m tall and 300 years old (Urrutia-Jalabert et al., 2018). Mean biomass was reported as 113.4 Mg C ha^-1^ while a biometric based estimate of net primary productivity was estimated to be 4.2 Mg C ha^-1^ year^-1^ (Urrutia-Jalabert et al., 2015a). Small patches of peatland dominated by the moss *Sphagnum spp*. and the dwarf conifer *Lepidothamnus fonckii* (Heusser, 1982, Barichivich, 2005) occur towards the eastern side of the forest.

### 2.2 Eddy covariance measurements and corrections

A closed-path eddy covariance (EC) system (CPEC200; Campbell Scientific Inc., Logan, USA; hereafter CSI) was installed on top of a 36 m tower to measure the net ecosystem exchange (NEE, μmol CO_2_ m^-2^ s^-1^) of the forest. The system is composed of an infrared gas analyzer (EC155; CSI) and a 3-D sonic anemometer (CSAT3A; CSI) that measures wind speed (m s^-1^) at 10 Hz. Additionally, a suite of micrometeorological variables are measured and stored every 30 minutes, including photosynthetically active radiation (PAR; μmol photon m^-2^ s^-1^; LI190SB, LI-COR, Lincoln, USA, hereafter LI-COR), air temperature (Ta, °C) and relative humidity (RH, %, HMP155A, Vaisala, Helsinki, Finland), net radiation (Rn; W m^-2^; NR Lite2, Kipp & Zonen, Delft, Netherlands), precipitation (Pp; mm; 52203 RM Young, Traverse City, USA) and soil heat flux (SHF; W m^-2^; three HFP01, Hukseflux, Delft, Netherlands). From these variables we estimated vapor pressure deficit (VPD; kPa), friction velocity (u*; m^-2^ s^-1^), and the relative extractable water for plants between the local wilting point and field capacity (REW).

We analyzed the first three years of data: Year 1, 02/2018 - 01/2019; Year 2, 02/2019 – 01-2020; Year 3, 02/2020 – 01/2021. Eddy-covariance data were processed and quality-controlled following three steps (see details in Supplementary Material Appendix 1): *i*) Biometeorological data from the EC system were pre-processed using a custom R script (R Core Team, 2020) that does the checking of the data for outliers (see supplementary material for thresholds) and a gap-filling using hourly data from the European Centre for Medium-Range Weather Forecasts (ECMWF) ERA5-Land Reanalysis (Muñoz-Sabater et al., 2019). This gap-filling is based on the Quantile Mapping approach (QMAP) and was done using the nearest grid point from ERA5 to infill values of Ta, RH, Pp. Global radiation (Rg; W m-2) was obtained from ERA5 using QMAP to be used as an input for the software EddyPro in the next step. *ii*) Raw eddy-covariance data were then processed with the EddyPro flux processing package (v. 7.0.6, Fratini and Mauder, 2014; LI-COR Biosciences, 2017) using the gap-filled biometeorology data to perform flux computations and corrections including Tilt Correction (Wilczak et al., 2001), Spectral Corrections (Moncrieff et al, 1997; Moncrieff et al, 2004), QC Flagging (Mauder and Folken, 2006), and footprint estimation (Kljun et al, 2004). *iii*) The final step to compute the final half-hourly fluxes is a post-processing using the REddyProc package (Wutzler et al., 2018) to perform quality correction of CO_2_ and the turbulent components of the energy balance, including the following workflow:

- Footprint correction to discard data when the highest contribution to turbulent fluxes (x_peak from EddyPro output variables) includes an area outside the ecosystem fetch.
- Data screening to eliminate NEE, LE and H data values that fall outside the mean ± 3 MAD range (median absolute deviation).
- u* filtering using a relation between fluxes and friction velocity to remove data when not enough turbulence is present, i.e., data that were measured under a possible influence of advection are removed (Reichstein et al., 2005; Papale et al, 2006).
- Gap-filling of fluxes after filtering by the criteria explained in points a-c. From a potential of 52,690 30-min data points in the study period, NEE, LE and H remaining data represented 44.1%, 43.7% and 46.8%, respectively. Gap-filling was performed using Marginal Distribution Sampling (MDS), which is based on the relationship between fluxes and environmental data (Reichstein et al., 2005).
- Partitioning of NEE data into gross primary productivity (GPP) and ecosystem respiration (Reco) using a flux partition procedure based on the night-time approach (Reichstein et al., 2005).

### 2.3 Soil respiration measurements and corrections

Soil respiration (Rs, μmol CO_2_ m^-2^ s^-1^) fluxes were measured around the EC tower with an automated soil CO_2_ flux system (model LI-8100A, LI-COR), connected to a multiplexer (model LI-8150, LI-COR) and four 20-cm diameter closed chambers (model LI-8100-104, LI-COR).

The chambers were installed over a PVC collar buried into the soil, which stayed in place during the whole sampling period (from July 3, 2019, to November 10, 2020) and were kept free of photosynthetically active material. The four chambers were installed within 15-m of the flux tower, sampling two conditions of the forest, namely under tree cover and in small forest gaps.

Rs was measured during 2 minutes in each chamber every 6 hours (following the recommendation by Perez-Quezada et al., 2016). To estimate the annual flux, we used the August 5, 2019, through August 4, 2020. Fluxes were estimated and screened for errors using the SoilFluxPro software from LI-COR Inc. Soil temperature (Ts, °C) and soil water content (SWC; v/v) were monitored at 5 cm depth with four sensors, close to each soil chamber, using thermocouple probes (TCAV, CSI) and water content reflectometers (CS616, CSI).

### 2.4 Estimation of environmental drivers and thresholds

Leading environmental drivers and their thresholds for NEE and Rs were determined using classification and regression trees (CART; Zhu et al., 2020). This technique explains a response variable from a set of predictor variables. Data splits (or so-called ‘leaves’ in the CART approach) that most significantly separate the means are created by minimizing the sums of squares within groups (De’ath and Fabricius, 2000). In our analysis, daily NEE was the response variable, and the predictor variables were daily (minimum, maximum and mean) Ta, PAR, Rg, Rn, Ts, SWC, RH, horizontal wind speed, VPD and u*; daily sums of Pp and daytime-PAR. Out of the 1096 days of analysis, 41 were deleted due to missing data. The analysis was also conducted for daily Rs using mean Ts, SWC, and the cover type where each chamber was placed (either below-tree-cover or forest gap) as predictor variables.

The variables selected by the analysis to make the splits corresponded to the leading drivers and the values of the splits defined the relevant thresholds. We set the maximum number of splits to 10 and the proportional reduction in error (PRE) to 0.03. CART analyses were carried out using the software SYSTAT 13.2 (Systat Software Inc., USA). To analyze the relationship between the C fluxes and the selected drivers, we fitted and plotted quadratic models for NEE, Rs, using the software SigmaPlot 14 (Systat Software Inc., USA). We also fitted quadratic models for GPP and Reco to analyze whether the drivers affected them differently.

### 2.5 Spatial representativeness of the productivity signal measured in the flux tower

We used satellite-derived solar-induced chlorophyll fluorescence (SIF) to investigate the spatial footprint of the local canopy productivity signal measured in our flux tower. SIF is a biophysical signal that results from the natural emission of photons by chlorophyll from the light-harvesting structures of plants during the process of photosynthesis (Baker, 2008). Remotely sensed SIF has been shown to have a strong positive linear relationship with canopy GPP, as observed at flux sites for temporal composites, and to track GPP seasonality better than traditional vegetation indices (Li et al., 2018; Zuromski et al., 2018; Magney et al., 2020). Recent advancements in Earth observation satellites have enabled global observation of SIF variability with an unprecedented spatial resolution (Sun et al., 2018) and now with a near-daily global coverage (Köhler et al., 2018; Guanter et al., 2021). Satellite-based SIF observations have been typically used to investigate the spatial and temporal patterns of the GPP of different ecosystems (Frankenberg et al., 2011; Sun et al., 2017).

We derived 8-day/0.2-degree gridded SIF data at 740 nm from corrected (sif_cor) daily adjusted Level-2B TROPOSIF retrievals from the 743-758 nm fitting window, from May 1, 2018 to February 1, 2021 (Guanter et al., 2021; available at https://s5p-troposif.noveltis.fr/data-access/). This dataset is based on the Tropospheric Monitoring Instrument (TROPOMI) aboard the Copernicus Sentinel-5P mission, which has a daily global continuous spatial sampling at 3.5 × 5.5 km^2^ resolution. The weekly temporal aggregation strengthens the linear relationship between SIF and GPP (Zhang et al., 2016) and also reduces the impact of the errors associated to individual SIF retrievals. Daily valid retrievals with cloud fraction <0.8 for the rainforest region of southern Chile were extracted and averaged to 8-day composite values in each 0.2° grid box. The same temporal averaging was done for the flux tower GPP observations. A correlation map between 8-day GPP variability at the flux tower and 8-day SIF variability at each grid box of the domain was then produced to assess the regional representativeness of the canopy productivity signal of the *Fitzroya* forest.

## 3 Results

### 3.1 Seasonal variation of microclimatic variables and carbon fluxes

**Figure 2:**
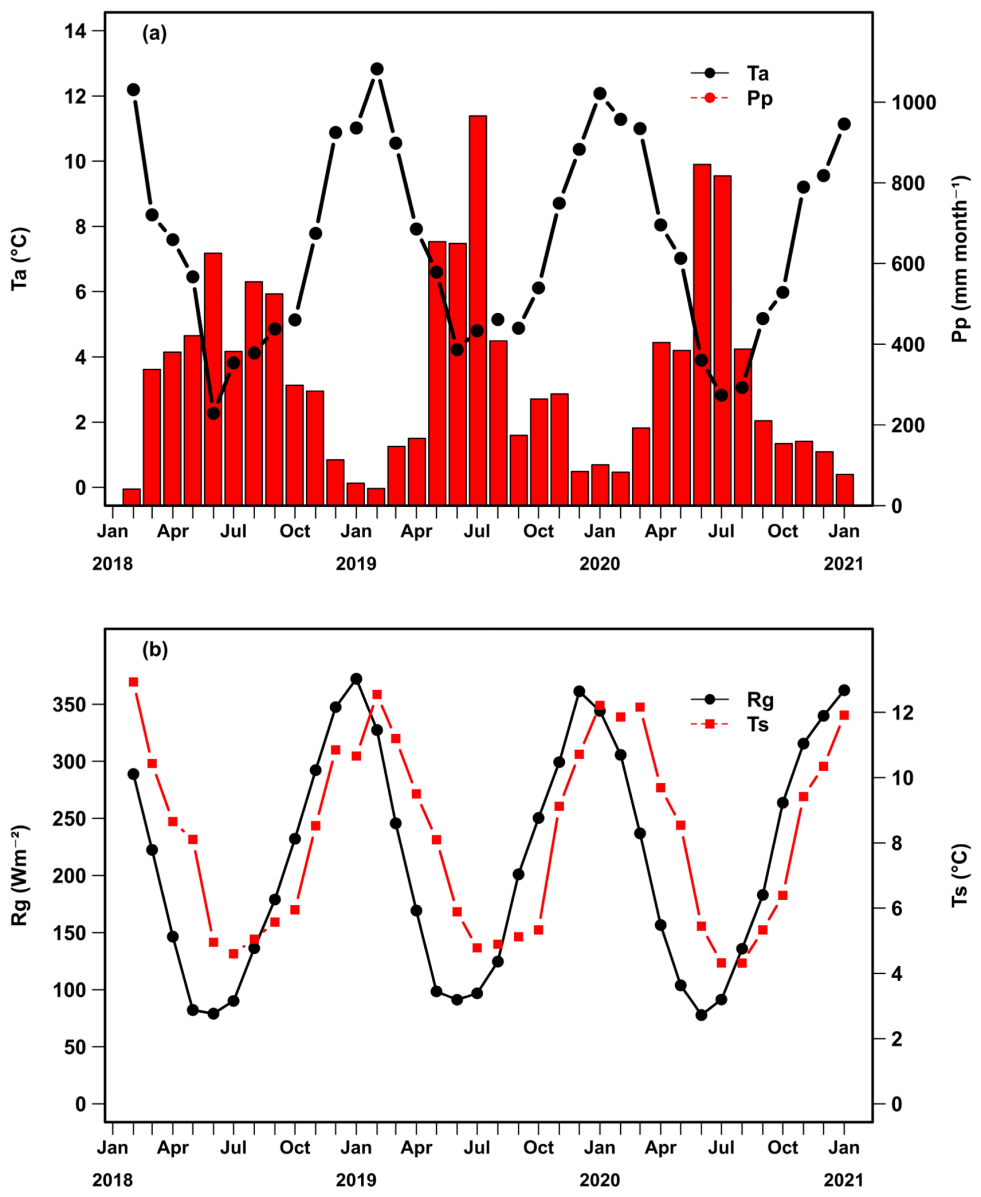
Monthly variation during the study period of a) air temperature (Ta) and precipitation (Pp), b) global radiation (Rg) and soil temperature (Ts), measured at the Alerce Costero Forest site (CL-ACF).

Mean annual Ta during the studied period varied between 7.0 °C and 7.8 °C, while observed precipitation varied between 3835 mm and 4018 mm, during the three study years (Table 1). There was a large variation in Pp and Ta between austral winter (June-August) and summer (December-February, Figure 2). Precipitation in winter months represented between 38 and 50% of annual precipitation, summer precipitation represented only 5-7%. The driest months within the recorded period were February 2018 and January-February 2019, and the latter recorded the highest mean temperature of the period.

**Figure 3:**
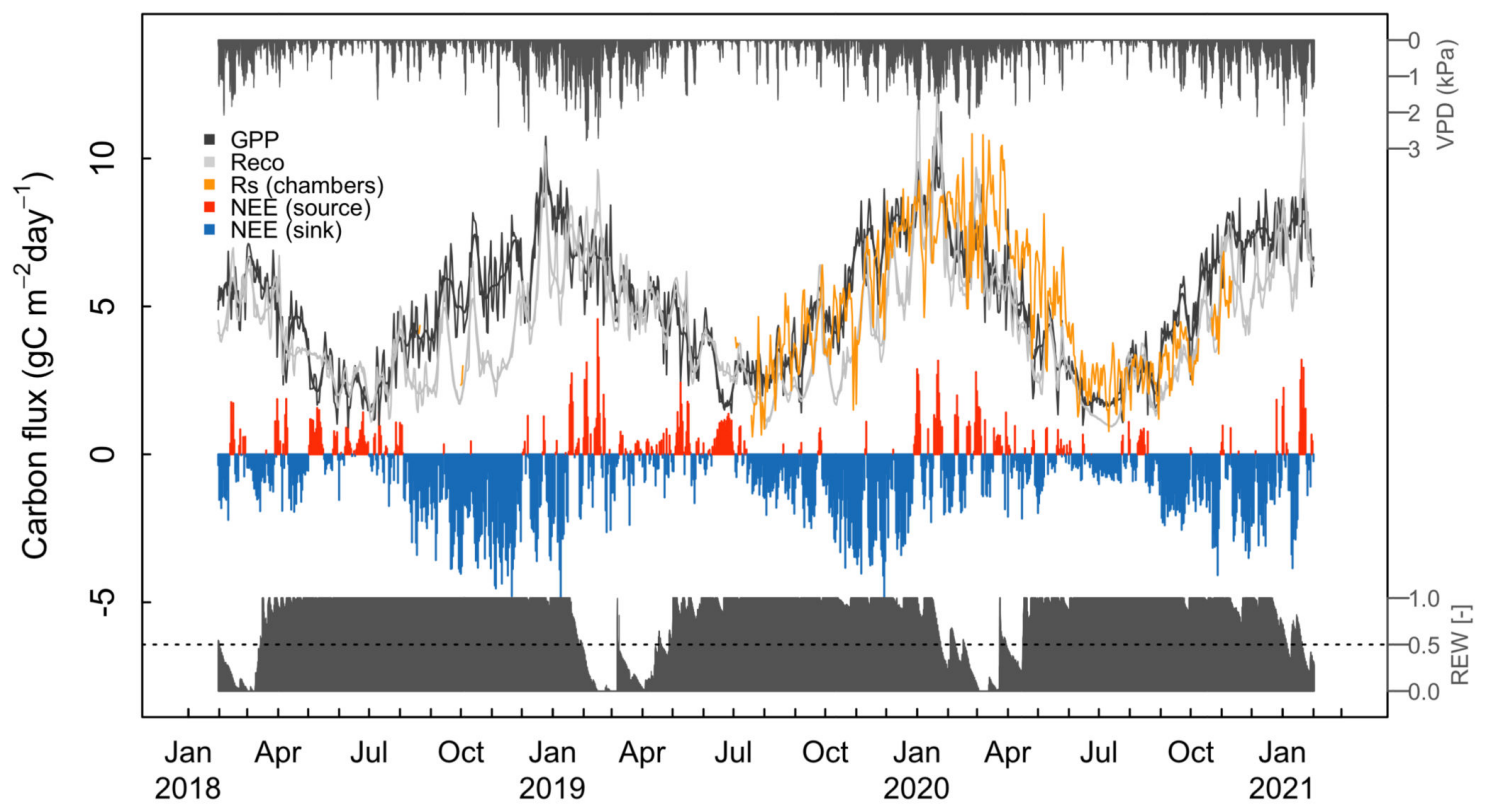
Seasonal variation of carbon fluxes along with atmospheric and soil dryness during the 3-year study period. The fluxes are daily gross primary productivity (GPP; black line), ecosystem respiration (Reco; light gray line), net ecosystem exchange (NEE; red and blue bars) and soil respiration (Rs; orange line). The thick lines for GPP and Reco depict a 15-day running mean. Vapor pressure deficit (VPD; inverted dark gray bars in the top) and relative extractable water for plants between the wilting point and field capacity (REW; dark gray bars in the bottom) represent atmospheric and soil dryness, respectively. Daily NEE is depicted as red or blue when the system behaves as a source or sink, respectively.

The seasonal variation of the ecosystem C fluxes showed that the forest acted as a C sink (negative NEE) most of the year and that net C emissions (positive NEE) occurred mainly in the summer months, a period that also showed the highest atmospheric (VPD) and soil dryness (Figure 3). The REW showed that during most of the austral winter and spring the upper layer of the soil profile is saturated whilst it dries up quickly in mid-January until mid-April (Figure 3), thus delineating a recurrent period of potential drought stress for this rainforest. NEE reached its maximum sink strength (~ -5 g C m^-2^ day^-1^) in spring, around November under cool and moist conditions but with high incoming radiation. Both GPP and Reco fluxes peak in summer, around January, after which they both become very similar. Rs fluxes follow a similar pattern compared to Reco, although it reaches a peak later in the summer (Figure 3).

**Figure 4:**
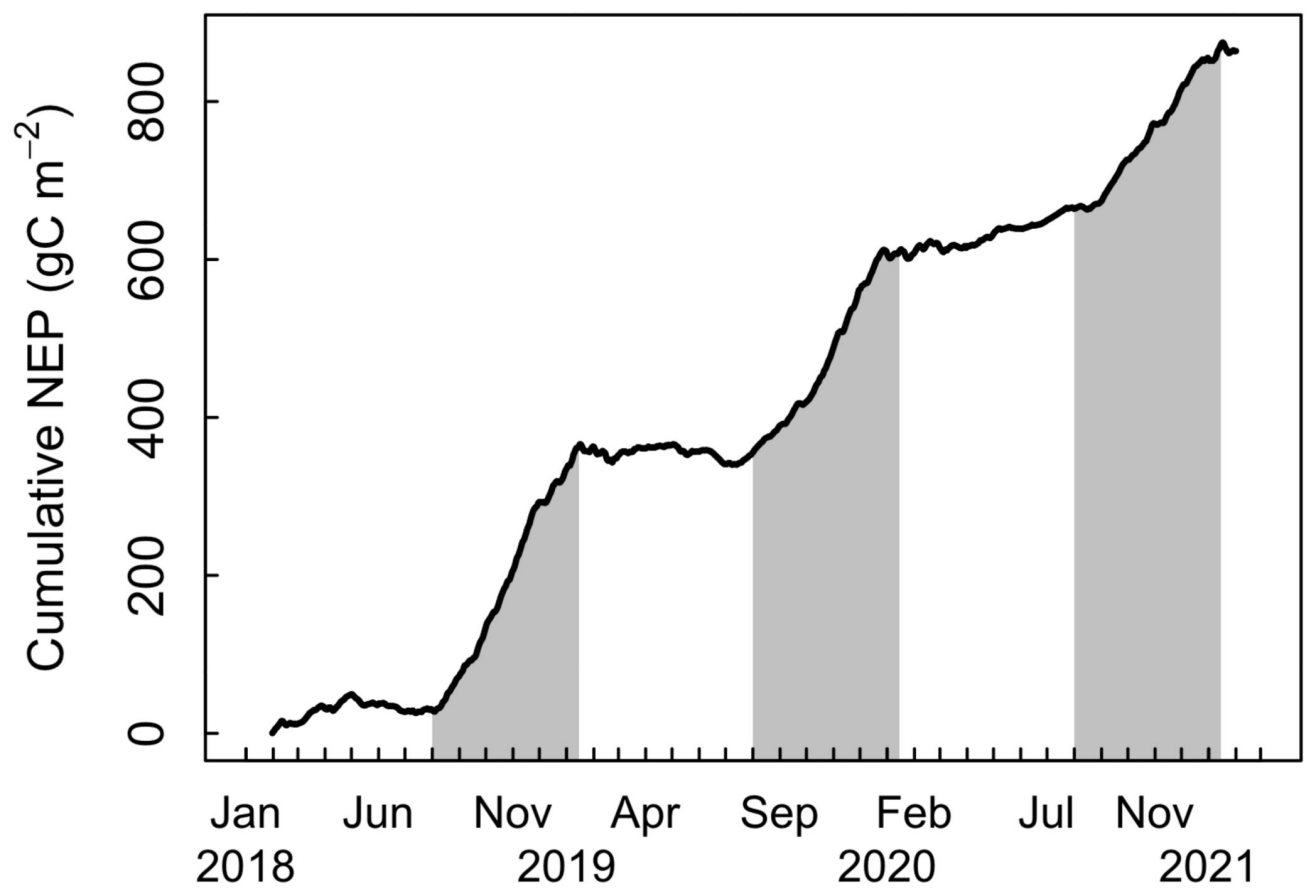
Cumulative net ecosystem productivity (NEP, i.e., the sum of NEE with inverted sign) to represent the carbon accumulation in the forest. The gray shading denotes the rough duration of the Carbon Uptake Period (CUP) from August to mid-January, after which the forest typically switches to a neutral carbon balance until midwinter in July.

Annual NEE varied between -245 and -362 g C m^-2^ year ^-1^, representing a moderate but persistent net C sink for all the three study years (-287±38 g C m^-2^ year ^-1^; Table 1), which is the result of mean GPP and Reco values of 1850 g C m^-2^ year ^-1^ and 1563 g C m^-2^ year ^-1^, respectively. The highest annual NEE was related to the lowest absolute values of GPP and Reco, and to lowest mean annual temperature (Table 1). Rs was more than twice higher when measured under tree cover (2077 g C m^-2^ year ^-1^) compared to that measured in forest gaps (938 g C m^-2^ year ^-1^), and the forest mean (1508 ± 366 g C m^-2^ year ^-1^) represented 96.5% of Reco (Table 1). The evolution of cumulative NEE shows that the Carbon Uptake Period (CUP) in the forest extends roughly through spring and mid-summer, lasting about 5 months between August and mid-January (Figure 4). The end of the CUP in mid-summer fluctuates between early and late January and occurs as a rapid switch to a period of near neutral carbon balance, which lasts by about 6-7 months until the end of July in mid-winter; in some years, there can still be a small net sink through winter (Figure 4).

### 3.2 Environmental drivers and thresholds of fluxes

**Figure 5:**
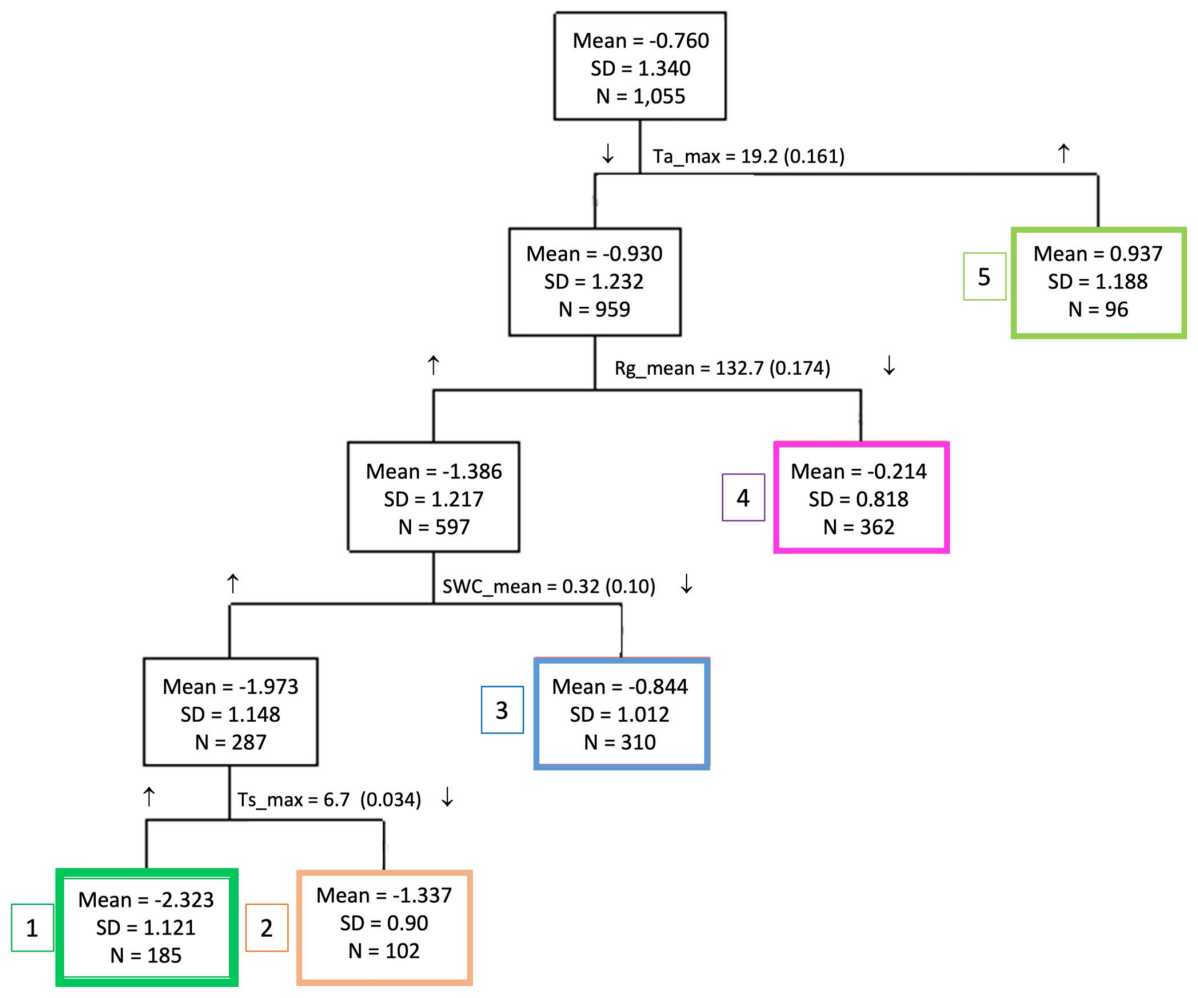
Classification tree derived from CART for daily NEE (g C m-2 day-1) with the most relevant environmental variables: Tamax, maximum air temperature (°C); Rgmean, mean global radiation (W m-2); SWC mean, mean soil water content (v/v); Tsmax, maximum soil temperature (°C). Boxes represent the five end-groups (leaves) and contain the mean, standard deviation, and number of NEE data, separated by relevant environmental variables and their corresponding thresholds. Arrows show whether an end-group was above (↑) or below (↓) the threshold. Values in parenthesis close to the thresholds represent the improvement in proportional reduction in error with each split.

**Figure 6:**
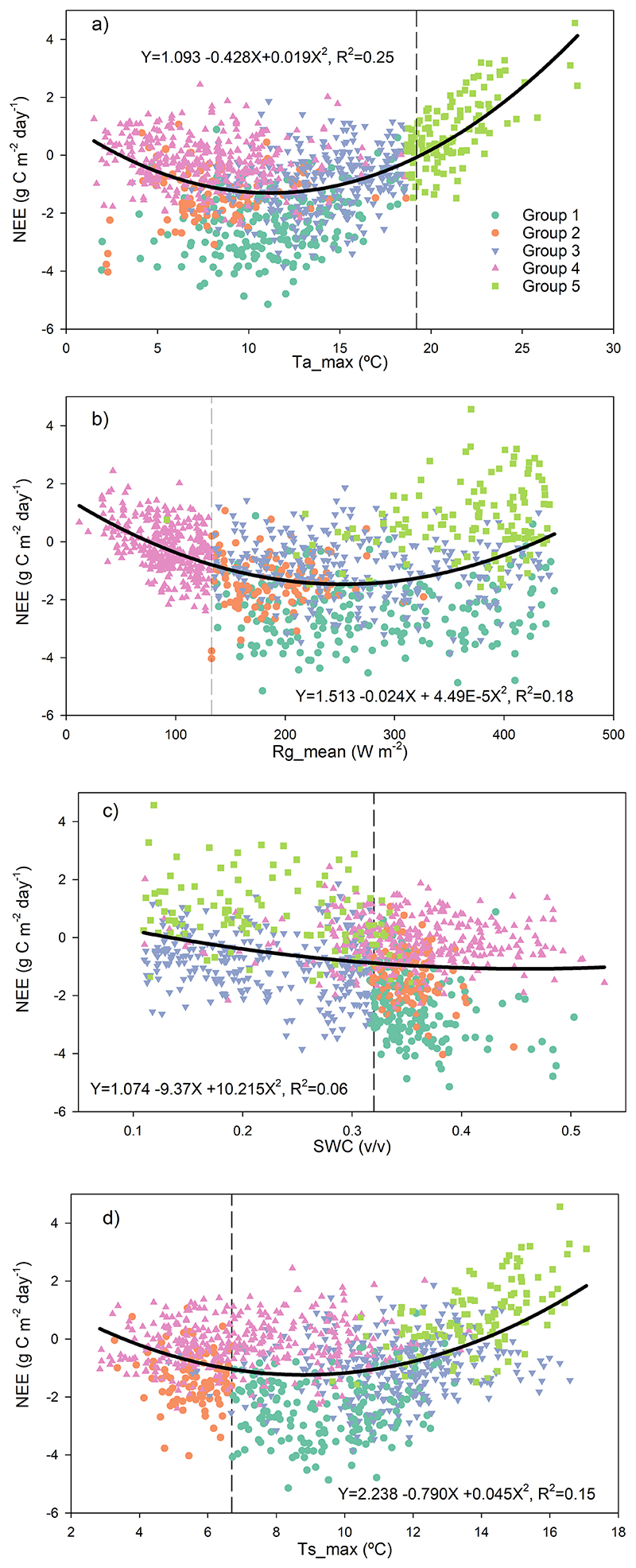
Effects of relevant environmental variables on daily-NEE. Tamax, maximum air temperature (°C); Rgmean, mean global radiation (W m-2); SWCmean, mean soil water content (v/v); Tsmax, maximum soil temperature (°C). Solid lines are quadratic models fit to the data; equations and coefficients of determination are included in each panel. Dashed vertical lines show the thresholds defined by each environmental variable, while colors represent the end-groups (leaves) defined by CART, shown in Figure 5.

**Figure 7:**
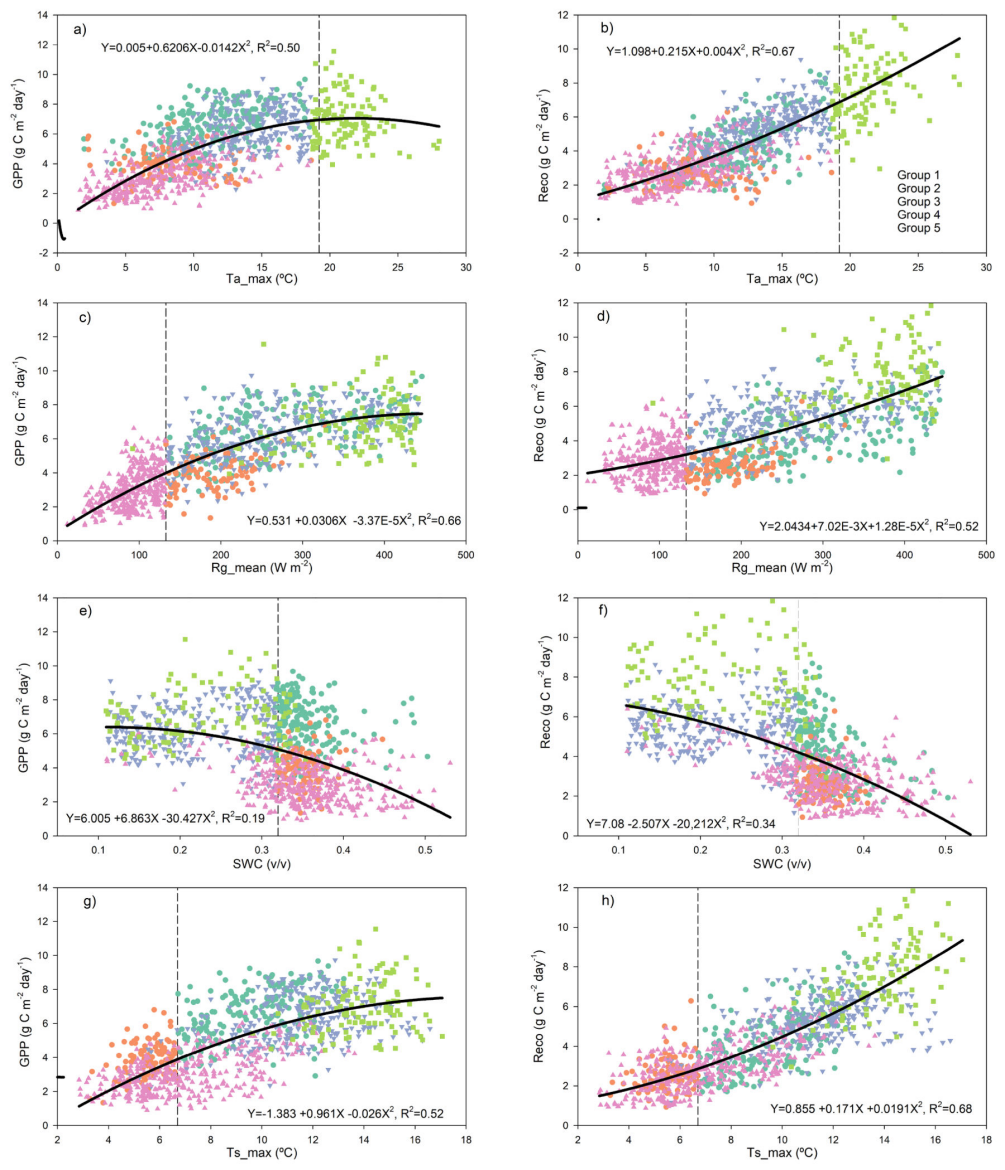
Effects of relevant environmental variables on daily GPP (left plots) and Reco (right plots) both in (μmol CO_2_ m-2 s-1). Tamax, maximum air temperature (°C); Rgmean, mean global radiation (W m-2); SWCmean, mean soil water content at 5 cm depth (v/v); Tsmax, maximum soil temperature at 5 cm depth (°C). Solid lines are quadratic models fit to the data; equations and coefficients of determination are included in each plot. Dashed vertical lines show the thresholds, while colors represent the end-groups (leaves) defined by CART for NEE, shown in Figure 5.

The most relevant environmental drivers for NEE were Ta_max_, Rg_mean_, SWC_mean_ and Ts_max_ (Figure 5). The total model proportional reduction in error (PRE) was 0.47 and separated five end-groups that differed in mean NEE, ranging from a sink of -2.2 μmol CO_2_ m^-2^ s^-1^ (Group 1) to a source of 0.9 μmol CO_2_ m^-2^ s^-1^ (Group 5). The highest C fixation occurred when Ta_max_<19.2 °C, Rg_mean_ <132.7 W m^-2^), SWC >0.32 and Ts_max_>6.7 (Group 1, Figure 5), conditions that occur primarily during austral spring (October-November, Figure 3). The largest C emission occurred when Ta_max_>19.2 °C (Group 5, Figure 5), which occurs during the summer (January-February). The Ta_max_ threshold marks the point above which the ecosystem became a C source (Figure 6a), because GPP decreased while Reco kept increasing (Figure 7a-b).

The threshold defined for daily Rg_mean_ (132.7 W m^-2^) was associated with the separation of Group 4 (Figure 6b), which contained values of NEE close to neutral that occurred mainly between May and August. Below this threshold, GPP grew actively (Figure 7c) but was counterbalanced by Reco that showed larger values at low radiation (represented by the higher intercept in Figure 7d). Mean daily Rs was mainly discriminated by Ts (above Ts= 8.5 °C Rs increased more steeply) and then by the type of cover (Rs was higher under tree cover compared to open spaces (Supplementary Material, Figure S1).

### 3.3 Representativeness of forest productivity measured in the flux tower

**Figure 8:**
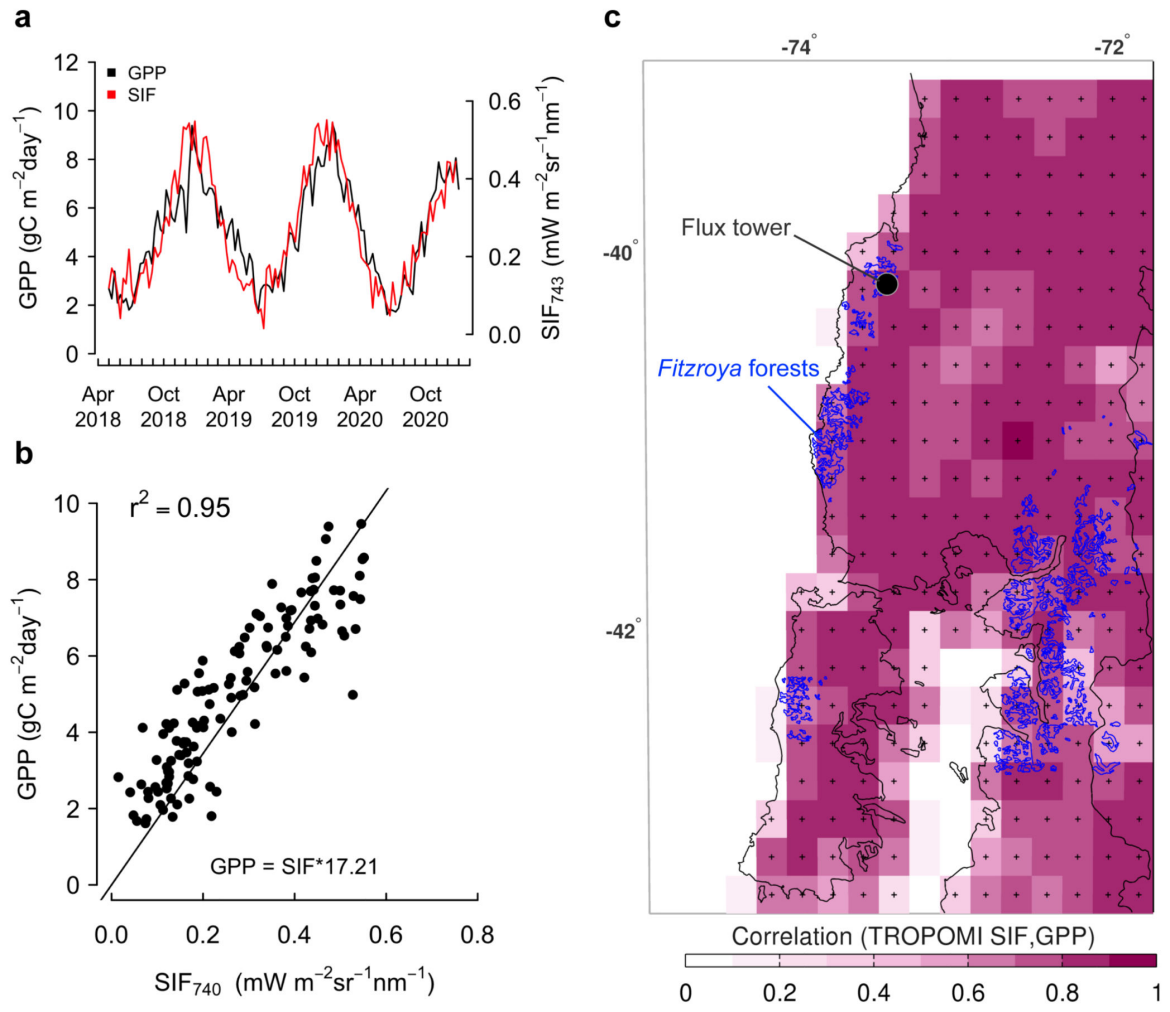
Comparison between 8-day GPP variability at the Fitzroya forest flux tower and TROPOMI-based sun-induced fluorescence (SIF) gridded at 0.2° from May 2018 to February 2021. a) Seasonal variation of the flux tower GPP (black) and SIF740 (red) at 8-day resolution. b) Linear fit with zero intercept between GPP and SIF740, b) and c) SIF over the nearest 0.2° grid cell from the Fitzroya flux tower. c) Correlation map between 8-day GPP variability at the flux tower and 0.2° gridded SIF740 over southern Chile. The “+” sign denotes significant correlations at the 95% confidence level.

Weekly GPP over the footprint of the tower are highly correlated with TROPOMI-based SIF data of the nearest 0.2-degree grid cell to the tower, with SIF closely following the seasonal variability of GPP (Figure 8a-b). Indeed, SIF explains 95% of the weekly GPP variability when using a linear fit forced through the origin (GPP = SIF*17.21, r^2^=0.95, *p* < 0.001). Although inferred at a broader spatial scale, the seasonal course of SIF data is remarkably in phase with that of the *in situ* GPP data. A correlation map between GPP data at the tower and gridded SIF over a large nearby area demonstrates that the strong GPP-SIF relationship found at the local scale also holds (r>0.6, *p*<0.05) with the cells that are mainly covered with *Fitzroya* (Figure 8c) and other temperate broadleaved evergreen native rainforests in south-central Chile between 39° and 43° S (Figure 1).

## 4 Discussion

### 4.1 A carbon sink of a regrowing rainforest adapted to cool conditions

Forest age and historical land use are major factors driving the rate of carbon uptake in a forest ecosystem (Finzi et al., 2020). As expected, the overall net carbon accumulation trend found in our mature *Fitzroya* forest (Figure 4) is consistent with its ongoing recovery from past fires and logging disturbance through vigorous growth and forest regeneration (Urrutia et al., 2015b). Low-intensity fires released the survivor trees from competition by neighbouring trees that were killed by the fires. In addition, abundant establishment of recruits of *Fitzroya* and other species has taken place under the semi-open canopy after the fires (Lara et al., 1999). Therefore, the growth increase of surviving trees and tree recruitment determined the development of a mosaic of fully stocked mature stands with some patches of vigorous regeneration (Lara et al., 1999). These processes in the footprint area of the eddy tower explain the increasing trend in the carbon sink documented for the *Fitzroya* forest in this study (NEE=-287±38 g C m^-2^ year^-1^). The current rate of carbon accumulation should continue into the near future in absence of disturbances until the canopy closes or climate constraints offset annual carbon gains. The observed NEE is 40% lower than the global average of NEE for temperate humid evergreen forests (-398 ± 42 g C m^-2^ year^-1^, Luyssaert et al., 2007). However, it is very similar to the NEE reported for a coastal Douglas-fir forest in Canada (-258 ±56 g C m^-2^ year^-1^; Jassal et al., 2007), which has similar mean temperature (8.5 °C) but much lower annual precipitation (1450 mm). Even GPP and Reco values were very similar (1815 ± 70 and 1557 ± 89 g C m^-2^ year^-1^, respectively) to the Douglas-fir forest, although Rs was much higher compared to their estimation (981 ±43 g C m^-2^ year^-1^). Annual NEE was 20% higher than that measured in an old-growth evergreen broadleaved North- Patagonian rainforest in southern Chile (-238 ± 31 g C m^-2^ year^-1^; Perez-Quezada et al., 2018) and 70% higher than in an open Mediterranean shrubland in central Chile (-82 ± 41 g C m^-2^ year^-1^; Meza et al., 2018).

The carbon uptake period of the *Fitzroya* rainforest starts in late austral winter, peaks in late spring and ends in midsummer and this pattern seems very stable through the years (Figure 4). This contrasts with the typical seasonal pattern of carbon uptake of temperate evergreen coniferous forests in the Northern Hemisphere, where net carbon uptake starts in spring, peaks in summer and ends in autumn (Richardson et al., 2010). A broadleaved rainforest located 180 km south of the Alerce Costero site has a similar starting date and peak for carbon uptake, but it ends in late autumn (Perez-Quezada et al., 2018). The distinctive seasonal dynamics of the *Fitzroya* rainforest shows that it is particularly adapted to cool and wet spring conditions and that it is strongly constrained by summer drought and warmth in the second half of the growing season. Unlike many cool Northern Hemisphere temperate forests, the oceanic influence on climate in our study site buffers it against freezing winter temperatures and despite its high elevation no persistent snowpack forms. Mostly snow-free conditions and temperatures well above freezing in winter therefore allow ecosystem metabolic activity to increase after the winter solstice along with increasing radiation and day length, without constraints from spring snowmelt or soil thaw as in cool mountain regions of the Northern Hemisphere. It is then the summer drought and warmth that constrain the end of the period of carbon uptake because of water shortage due to shallow soils and rainless conditions. This results in a shorter period of carbon uptake than in a typical temperate evergreen forest in the Northern Hemisphere and consequently lower annual NEE magnitude compared with the global NEE average of temperate evergreen forests (398 ± 42 g C m^-2^ year ^-1^; Luyssaert et al., 2007). However, annual GPP and Reco are higher compared to the global average (1762 ± 56 g C m^-2^ year ^-1^; and 1336 ± 57 g C m^-2^ year ^-1^, respectively; Luyssaert et al., 2007), which agrees with what was reported for an evergreen broadleaved rainforest in southern Chile (Perez-Quezada et al., 2018).

### 4.2 Dry and warm conditions weaken the carbon sink

Our flux measurements revealed that the *Fitzroya* rainforest ecosystem thrives under cool, moist environmental conditions. Photosynthesis occurs year-round and GPP consistently exceeds Reco for nearly half of the year between July and January (Figure 3). Among the leading biophysical relationships, we found that the net carbon sink of the ecosystem is maximal when daily Ta_max_ is about 11 °C (Figure 6a), which relates to daily mean air temperatures that are similar to the mean annual temperature around 7 °C in October-November but weakens as temperatures increase above this value. The C balance of the forest is affected when maximum temperature rises above 19°C (Figure 6a) because photosynthesis starts to decrease whilst C loses to respiration keep increasing (Figures 7a and b). This is in line with what has been reported for the species before, being maximum temperature the main climatic variable negatively affecting the radial growth of *Fitzroya* (Urrutia-Jalabert et al., 2015b, 2015c, Lara et al., 2020). In fact, it was previously reported that warm days, and their associated high vapor pressure deficits, would reduce the number of days with adequate environmental conditions for growth to occur (Urrutia-Jalabert et al., 2015b). Moreover, high radiation values were also reported to negatively affect radial growth in trees from this site (Urrutia-Jalabert et al., 2015b), which agrees with the negative effect of high radiation on C fluxes reported here. The particularly low thermal optimal of the carbon sink of *Fitzroya* at maximum air temperatures around 11°C is (Figure 6 a) consistent with the idea of a relict Southern Hemisphere conifer better adapted to past colder climates (Enright and Hill, 1995; Ahuja 2017), a view of the species long held by paleo biogeographers based on fossil pollen analyses (Heusser et al., 1982).

This pattern of temperature response interacts with soil water availability. The forest turns into a near neutral carbon balance when soil water content decreases below 30% during the summer dry season, because both photosynthesis and ecosystem respiration increase at the same pace as the soil dries (Figure 7 e-f). This shows that even though annual precipitation in this site is high (>4000 mm), the low water retention capacity of the soil plays a major role in the forest water and carbon balances. It has indeed been reported that the low water retention capacity of the soils in this site has been critical for the negative radial growth trend of the forest since the 1970s, which has not been seen in many sites of the Andean Cordillera (Urrutia-Jalabert et al., 2015c, Lara et al., 2020). Moreover, low soil water content was also reported to drive high soil respiration in this site (Urrutia-Jalabert et al., 2017).

The thresholds of relevant drivers that separated the end-groups of NEE (Figure 5) seemed related to their effect on GPP and Reco (Figure 7). For maximum temperature, mean global radiation and maximum soil temperature, the drivers showed different curve shapes for GPP compared to Reco; i.e., they caused an asymptotic response of GPP whereas Reco showed an exponential response (Figure 7). These results suggest that warming will disproportionately increase Reco compared with GPP during the cold season and will also directly reduce photosynthesis during the increasingly warm and dry summer season. Furthermore, intensified drought and heat stress in spring- early summer will reduce radial growth and carbon sequestration in woody biomass. In fact, despite radial growth being reported to mainly occur between November and March in the area (i.e., clear stem daily cycles, Urrutia-Jalabert et al., 2015b), it has also been previously suggested that growth during spring may be even more important than summer growth at this site (Urrutia-Jalabert et al., 2020).

Annual soil respiration measured in our study was higher compared to previous monthly measurements using the manual closed chamber method in *Fitzroya* forests in 15 points systematically distributed in two 0.6 ha plots located around the tower (637-864 g C m^-2^ year^-1^ for years 2011-2012 and 2012-2013, respectively; Urrutia-Jalabert et al., 2017) although the main drivers (soil temperature and water content) are consistent in both studies. The main difference could be due to the different methods used, since in the previous study measurements were done using a manual environmental gas analyzer with a closed static chamber once a month (values were interpolated between measurements). The high fine root biomass reported for the *Fitzroya* forests surrounding the area of the tower and a higher proportion of autotrophic respiration, compared with heterotrophic respiration in this site (Urrutia-Jalabert et al., 2017), may explain the high difference observed between areas with tree cover and small forest gaps.

Annual soil respiration values obtained in the current study are similar to the ones reported for high biomass and old-growth temperate rainforests from Oregon (1080–2070 g C m^-2^ year^-1^; Campbell and Law 2005), and within the range of respiration values reported for temperate coniferous forests from the Northern Hemisphere (427–1805 g C cm^-2^ year^-1^; Hibbard et al. 2005).

Paleoendemic conifers with narrow ranges such as *Fitzroya* are particularly threatened in their native endemic locations in the face of rapid climate change (Jordan et al., 2016; Ahuja 2017). The environmental responses of the carbon sink of *Fitzroya* forests suggest that they will be negatively affected by projected warmer and drier conditions in the region with further intensification of climate change (Bozkurt et al., 2018; Boisier et al., 2018). The summer dry season has already perceptibly intensified and lengthened in recent years during the ongoing megadrought (Garreaud et al., 2017), with maximum daily air temperatures exceeding the normal envelope and increasingly rising up to 30°C. These extremes have already caused extensive canopy mortality of evergreen *Nothofagus* forests and isolated *Fitzroya* trees in the proximity of the flux tower (Barichivich, 2005), which also coincide with a gradual process that we have observed in the field occurring during the last decade. However, it is still not clear to what extent *Fitzroya* will be affected in its growth, carbon balance and survival under the projected warmer and drier conditions in the region. In a previous study, it was reported that the driest summers recorded in southern Chile (2014-2015, 2015-2016) did not produce a significant reduction in radial growth of *Fitzroya* at the Alerce Costero site, suggesting among other reasons, that spring climate conditions were probably more influential compared to those prevailing in the summer and/or that concurrent radial growth carbon demands are not that high at this low-productivity site (Urrutia-Jalabert et al., 2020). Moreover, vulnerability to dry conditions seems to differ at different tree development stages (Urrutia-Jalabert et al., 2018).

In the coming decades, the frequency and intensity of extreme summer droughts and heatwaves will continue increasing (Feron et al., 2019; Cook et al., 2020) to levels beyond the envelope of the cool-wet climate space of the species (Fig. 1c). According to the results documented in this study, it might be expected that the period of carbon uptake of the forest will shorten and the whole system will be pushed towards a net carbon source during summer and autumn, which might offset spring carbon gains. Warming in the rainy cool season is also occurring, but its magnitude is uncertain in this mountain region because of the lack of long *in situ* meteorological records. However, less frequent snowfalls in the summit of the Coastal Range together with land surface phenology signals of earlier increases in spring temperature (Chavez et al., 2021), suggest that winter and spring are experiencing warming despite a recent anomalous offshore cooling in the midlatitudes of the South American west coast (Falvey and Garreaud, 2009). Spring warming will likely enhance the rates of ecosystem respiration, reducing the peak of the seasonal carbon sink of *Fitzroya* around its cool optimal. Overall, drying and warming might weaken the annual carbon sink of *Fitzroya* by shortening the carbon uptake period, decreasing the magnitude of the net sink in spring and increasing the carbon losses in the current period of carbon neutrality in summer and autumn. Additional ecological and ecosystem modelling studies are needed to better predict future forest dynamics of this iconic and endangered paleoendemic species under intensifying climate change.

### 4.3 Coherent local and regional canopy productivity signal from the flux tower

The TROPOMI-based sun-induced fluorescence (SIF) data (at weekly/0.2° scales) was highly correlated with the canopy GPP signal estimated within the flux tower footprint and correlated well with the SIF retrieved in the region between 39° - 43°, particularly over areas covered by *Fitzroya* and other temperate broadleaved evergreen native rainforests in south-central Chile (r > 0.6; Figures 1 and 8c). Other areas covered by grasslands, shrublands and evergreen forest plantations showed lower correlation but still a significant relation because of an in-phase photosynthetic seasonality (Figures 1 and 8c). Therefore, variations in the productivity of the *Fitzroya* forest around the tower are representative of a coherent regional signal of canopy productivity variation.

The slope of the strong linear fit with zero intercept found between *Fitzroya* GPP and TROPOMI-SIF was 17.21 g C m^− 2^ d^−1^/ W m^−2^ *μ*m^−1^ sr^−1^ (Figure 8c). It is substantially higher than the convergent slope of ~13.5 g C m^− 2^ d^−1^/ W m^−2^ *μ*m^−1^ sr^−1^ found in Northern Hemisphere forests with the same dataset using a linear regression forced through the origin (Li and Xiao, 2022). Thus, it apparently does not support the view of a universal SIF-GPP relationship across different vegetation types (Sun et al., 2017; Li et al., 2018; Li and Xiao, 2022). This difference is not related to a scale mismatch between satellite and flux tower footprints because the slope of the SIF-GPP fit varies little with spatial aggregation of SIF (Li and Xiao, 2022). In our evergreen rainforest study site where there is no full winter dormancy, SIF values close to zero in late autumn and winter (May-August) are still associated with GPP values around 2 g C m^− 2^ d^−1^ around the tower (Figure 8b). Forcing the SIF-GPP linear regression through the origin at this site results in an underestimate of GPP in the cold season, although it is consistent with the theoretical expectation that leaf photosynthesis may be zero when the quantity of emitted SIF radiation is zero. However, this does not necessarily imply that the canopy level SIF-GPP relationship extends linearly to zero (Pickering et al., 2022). Indeed, it has been shown that the canopy level SIF-GPP relationship smooths over known non-linearities at lower SIF yields (Magney et al., 2020) and that the intercept in productive tropical evergreen broadleaved forests can be far from zero (Li et al., 2018). This means that forcing the linear regression of SIF-GPP through the origin based on a prior expectation (i.e., that SIF and GPP are simultaneously zero as in winter-dormant Northern Hemisphere forests) that lies outside the range of the data will introduce a bias into the regression parameters in this evergreen system that is active year-round (Pickering et al., 2022).

A best linear fit with free intercept (GPP=SIF*12.38 + 1.69; R^2^=0.79, p<0.001) in our site yields a slope of 12.38 g C m^− 2^ d^−1^/ W m^−2^ *μ*m^−1^ sr^−1^, which is closer to the ‘universal slope’ from a regression forced through the origin in seasonally dormant Northern Hemisphere forests based on TROPOMI SIF (Li and Xiao, 2022). Overall, our result suggests that SIF is suitable for monitoring evergreen rainforest photosynthesis (broadleaved and needleleaf) at the landscape scale across the region, but the evaluation of the global SIF-GPP relationship should consider a varying intercept to account for differences in the phenology of evergreen forests that can potentially photosynthesize year-round. This result is important because global modelling efforts acknowledge that current spatial biases of information towards the Northern Hemisphere influence current carbon cycle model parameterization and interpretation of ecosystem dynamics of underrepresented regions, such as the austral temperate rainforest biome (Stell et al., 2021).

### 4.4 Implications for national climate policy and outlook

Forest ecosystems play a pivotal role to reach carbon neutrality due to its capacity to capture and store carbon in their various pools (i.e., living and dead biomass in trunks, branches, roots) compensating for the emissions from fossil fuel combustion. The contemporary and future carbon sink and emissions from the 14.7 million hectares of native forests in Chile (CONAF 2022) is still very uncertain because of the lack of sufficient direct carbon flux measurements and limited modelling capacities and research infrastructure. Also, the increasing loss of native forests due to human set fires and forest conversion to shrublands, agriculture and forest plantations as well as drought-driven browning (canopy dieback) in central Chile since 2010 add to this uncertainty (Heilmayr et al., 2019, Miranda et al., 2019, 2020). In June 2022, Chile passed a climate change framework law with the goal of reaching carbon neutrality by 2050, in line with Nationally Determined Contributions (NDCs) committed at the COP25 in 2020 within the United Nations Climate Change Convention.

Our measurements and associated capacity building are therefore a valuable and timely contribution towards a better quantification of the carbon cycle of native forests to inform national climate policy in the near future. However, to advance towards this goal, the country must urgently invest in establishing a long-term network of flux measurements in long-term ecosystem study sites across different biomes and secure funding for its functioning. In the Southern Hemisphere, such national flux networks already exist for more than a decade in Brazil (Restrepo-Coupe et al., 2013), Australia and New Zealand (Beringer et al., 2016), while the ChileFlux network was recently established in 2022 with 6 long-term sites. Regional flux data will enable the development and validation of terrestrial biosphere models to upscale and inform the national assessment of terrestrial carbon and water balances. Future research based on these new flux observations together with other ecosystem data, will allow parameterizing a global land surface model (e.g., Barichivich et al., 2021) and forecast the future of the carbon sink capacity of these globally unique paleoendemic *Fitzroya* rainforests. The close relationship found between productivity and satellite-derived SIF at local and regional scales is encouraging for the assimilation of SIF observations (MacBean et al., 2018) to further constrain carbon cycle model parameterizations in the region, while the flux network grows.

## 5 Conclusions

Our flux measurements demonstrate that the capacity to capture C of the temperate *Fitzroya* rainforests in the summit of the Coastal Range of southern Chile is optimal under cool and rainy conditions in early austral spring (October-November) and decreases rapidly towards the summer dry season (January-February) and autumn, when carbon losses during hot and dry conditions offset or exceed carbon gains. The *Fitzroya* forests were a moderate but consistent C sink in all three years, but seem vulnerable to warming and drought, particularly to the effects of maximum air temperature above 19 °C. TROPOMI-based sun induced fluorescence is highly correlated at the local and regional scales with gross primary productivity measured at the flux tower, which implies that the continued monitoring of ecosystem fluxes in the study site should allow modelling the full response of these cold-adapted rainforests under different scenarios of climate change. This may allow us to anticipate the impacts of warming and intensifying summer drying during the coming decades, which can support decision making and policy measures. This is important considering the relevance that the conservation of these forests have in relation to both the climatic and biodiversity crises.

## Supplementary Material

Supplementary Figure

## Figures and Tables

**Table 1:** Annual values and mean (± standard error) of meteorological variables and C fluxes (g C m^-2^ year ^-1^)

	2018	2019	2020	Mean ± SE
Met. Var.				
Air temperature (°C)	7.0	7.8	7.3	7.4 ± 0.2
Precipitation (mm)	4018	3948	3835	3934 ± 53
C Flux				
NEE	-362	-245	-255	-287 ± 38
Reco	1442	1651	1595	1563 ± 62
GPP	1804	1896	1849	1850 ± 26
Rs				1508 ± 366
Rs-cover				2077 ± 31
Rs-open				938 ± 395

## Key Points

1. We present the first analysis of carbon fluxes of a paleoendemic temperate rainforest in a global drying hotspot.
2. The paleoendemic ecosystem thrives under cool and wet conditions during the austral spring.
3. Projected warming and drying in the region threaten the carbon sink capacity of this globally unique, ultra-long-lived rainforest.

## Plain Language Summary

Forest ecosystems play a central role in climate policy because their growth removes nearly a third of the anthropogenic carbon emissions to the atmosphere. Yet, warming and drying with climate change threaten the capacity of forests to absorb carbon in some regions. Measuring the carbon sink of forests and its climatic control is thus essential for ecosystem management and climate policy, but current measurements are overwhelmingly concentrated on the Northern Hemisphere leaving a gap in the Southern Hemisphere. Here, we present the first measurements of carbon fluxes of an adult 300-year-old temperate rainforest of the ultra-long-lived conifer *Fitzroya cupressoides* in southern Chile. This conifer is a paleoendemic tree confined to a few cool and very wet climate refugia. Our results show that the forest can photosynthesise year-round because it is particularly adapted to cold and rainy conditions. Photosynthesis consistently exceeds ecosystem carbon losses by respiration between July and January, resulting in a moderate carbon sink compared with temperate forests in the Northern Hemisphere. Summer drying and warming enhance carbon losses by respiration and thus weaken the carbon sink capacity, implying that the ongoing and future climate drying and warming in the region threaten the carbon sink of this unique rainforest.
