# Supplementary figures and images for: Warming and drought weaken the carbon sink capacity of an endangered paleoendemic temperate rainforest in South America
Source: J Geophys Res Biogeosci. Author manuscript; Available in PMC 2023 Jul 16. (PMC7614759; doi:10.1029/2022JG007258)

Figure S1

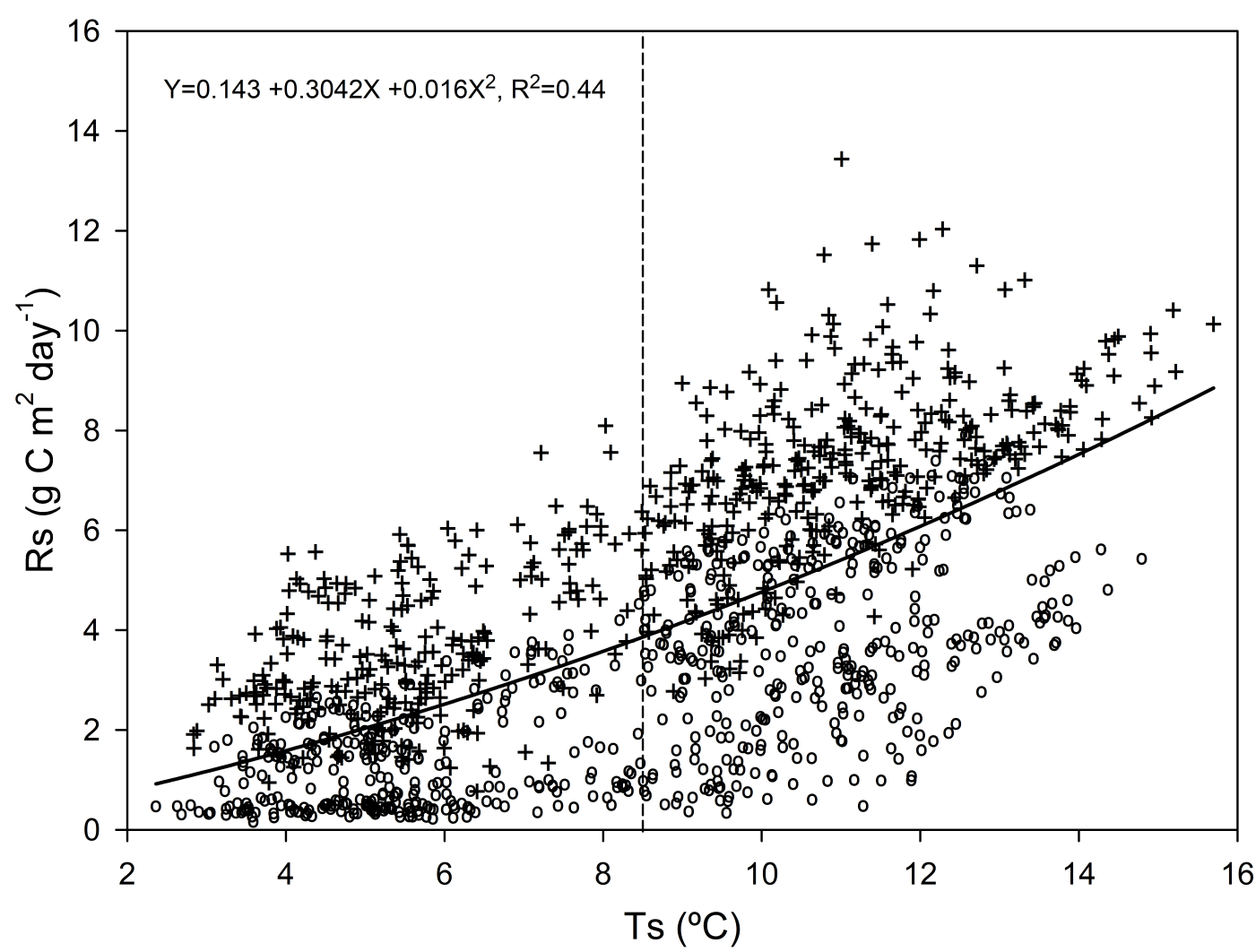

Supplement: Supplementary Figure [file EMS178538-supplement-Supplementary_Figure.pdf]
